# Structural investigation of Zungeru-Kalangai fault zone and its environ, Nigeria using aeromagnetic and remote sensing data

**DOI:** 10.1016/j.heliyon.2022.e09055

**Published:** 2022-03-05

**Authors:** A.B. Arogundade, M.O. Awoyemi, O.S. Hammed, S.C. Falade, O.D. Ajama

**Affiliations:** a Department of Physics and Engineering Physics, Obafemi Awolowo University, Ile-Ife, Nigeria; b Department of Physics, Federal University Oye-Ekiti, Nigeria; c Department of Physical Sciences, Landmark University Omu-Aran, Kwara State, Nigeria; d Ondo State Ministry of Education, Science and Technology, Nigeria

**Keywords:** Modelling, Subsurface structures, Remote sensing, Aeromagnetics, Fracture zones, Source edge detection

## Abstract

This study analysed aeromagnetic and satellite imagery data over parts of northern Nigeria to delineate magnetic and surface lineaments associated with fault system trends. The aeromagnetic data were analysed using the Fast Fourier transform technique for reduction-to-equator, total horizontal derivative, analytic signal amplitude and Euler deconvolution. Landsat-8 OLI and SRTM data have been enhanced using spatial filtering and hill-shading techniques for the delineation of lineament features. The extracted surface lineaments are denser on the satellite imageries than on the HRAD because the datasets respond to different physical properties of the geological units and features. The location and orientation of the Zungeru/Kalangai fault zone, which extends about 245 km from the Bida Basin Basement to the northern part of Nigeria, correlate with the existing fault on the published geological map and form a conjugate pair with a fault around Kaya. The derived maps revealed the presence of several previously undetected geological lineaments corresponding to faults and folded dyke, striking predominantly N–S, NNE-SSW, NE-SW and NW-SE lacking in previous geological maps. The application of 2D forward modelling revealed the 2D image of the nature of the subsurface, magnetic susceptibilities of rocks and the block boundaries coinciding with the geological lineaments.

## 1 Introduction

The Earth is a dynamic and changing system that is divided into several rigid blocks known as tectonic plates. The movements of the tectonic plates of the earth induce stress that is capable of deforming rocks. As an integral part of the West African Craton, the Precambrian Basement Complex of Nigeria has been subjected to various episodes of deformation (e.g. Tsalha et al., 2015; Awoyemi et al., 2017a). These deformations and metamorphism, which was formed in the Neoproterozoic, between 750 and 500 Ma, are considered to have resulted from an oblique collision of the Nigerian shield with the West African Craton (750-500 Ma) (Ferré et al., 2002; Kröner and Stern, 2005) and reflect the presence of geological structures of varying magnitudes. Furthermore, previous studies have shown that there is an inland extension of the oceanic fracture zones after offsetting the Mid-Atlantic Ridge and manifest as fault zones in the Nigerian Basement Complex (Wright, 1976; Whiteman, 1982; Binks and Fairhead, 1992; Adepelumi et al., 2008; Ajama et al., 2021). These fracture zones are believed to have created some megastructures (such as Ifewara and Zungeru-Kalangai fault zones) that serve as weaknesses zones within the Nigeria Basement (Anifowose et al., 2006; Kolawole and Anifowose, 2011; Awoyemi et al., 2017a).

Since structures are manifestations of basement tectonic activity, there has been a considerable interest in studying the Earth's subsurface linear features and their implications over the years. Structures analyses not only provide a method for detecting past tectonic trends but also aid in volcano-tectonic and magmatic studies, seismic risk assessment for nuclear sites, groundwater, mineral and oil exploration, and repository studies (e.g. Over et al., 2004; Mostafa and Bishta, 2005; Ranganai and Ebinger, 2008; Ajama et al., 2017; Olasunkanmi et al., 2020; Arogundade et al., 2022).

The Zungeru/Kalangai fault is not a localised feature but a shear zone that extends over hundreds of kilometres and is accompanied by many minor and major faults and folds. Numerous literature on the investigation of structural lineaments has been presented by various authors (e.g. Agbebia and Egesi, 2020; Oladejo et al., 2020; Ajama et al., 2021; Osagie et al., 2021; Salawu et al., 2021). However, few studies were carried out on the Zungeru/Kalangai fault segment (e.g., Truswell and Cope, 1963; McCurry, 1976; Kolawole and Anifowose, 2011; Abubakar, 2012; Abdul Malik et al., 2018). The geophysical evidence and structural attributes that will buttress the extension of the Zungeru-Kalangai fault zone from the Bida Basin Basement and reveal undetected faults need to be provided.

The present study, which builds on the findings of Awoyemi et al. (2017a and 2017b), involves the use of satellite imageries and high-resolution aeromagnetic data as a structural mapping tool for delineating structural features caused by the deformational episodes that the rocks in this study area experienced. The findings of this research will help to advance understanding of Nigeria's structural geology. It could also be useful for groundwater investigations, mineral prospecting, and geotechnical engineering assessments of the area.

## 2 Geological description of the study area

The study area, located in a part of Northern Nigeria, is bounded by Latitudes 9°30′ N to 11°30′ N and Longitudes 6^o^00′ E to 7°30′ E. It covers approximately 36,300 km^2^, spanning parts of Zamfara, Katsina, Niger and the Kaduna States. The geology of the area is underlain by the Kushaka and Birnin-Gwari schist formations (Obaje, 2009) which are fault-controlled rift-like structures (Olade and Elueze, 1979). These schist belts are enclosed in the Kusheriki schist group in northern Nigeria and are estimated to be in the Kibaran (1,159 ± 70 Ma). According to Turner (1983) and Obaje (2009), the Kusheriki schist group comprises Birnin-Gwari schist formation at the top of the succession, Zungeru granulite formation, Kushaka schist formation and Kusheriki psammite formation at the bottom. The schist belts are considered Upper Proterozoic supracrustal rocks that have been infolded into the migmatite-gneiss-quartzite complex (Agbor, 2014).

The Birnin-Gwari schist formation and the underlying rocks of the Zungeru granulite formation together form a single structural unit, termed the Zungeru-Birnin Gwari schist belt (Ajibade et al., 2008; Obaje, 2009). The Birnin-Gwari schist formation is flanked with Zungeru mylonites on both sides and strike the western side of the Kushaka schist belts. The Birnin Gwari formation includes phyllites, schists, and schistose mudstone conglomerates and occupies the synclinal axis of the schist belt.

**Figure 1:**
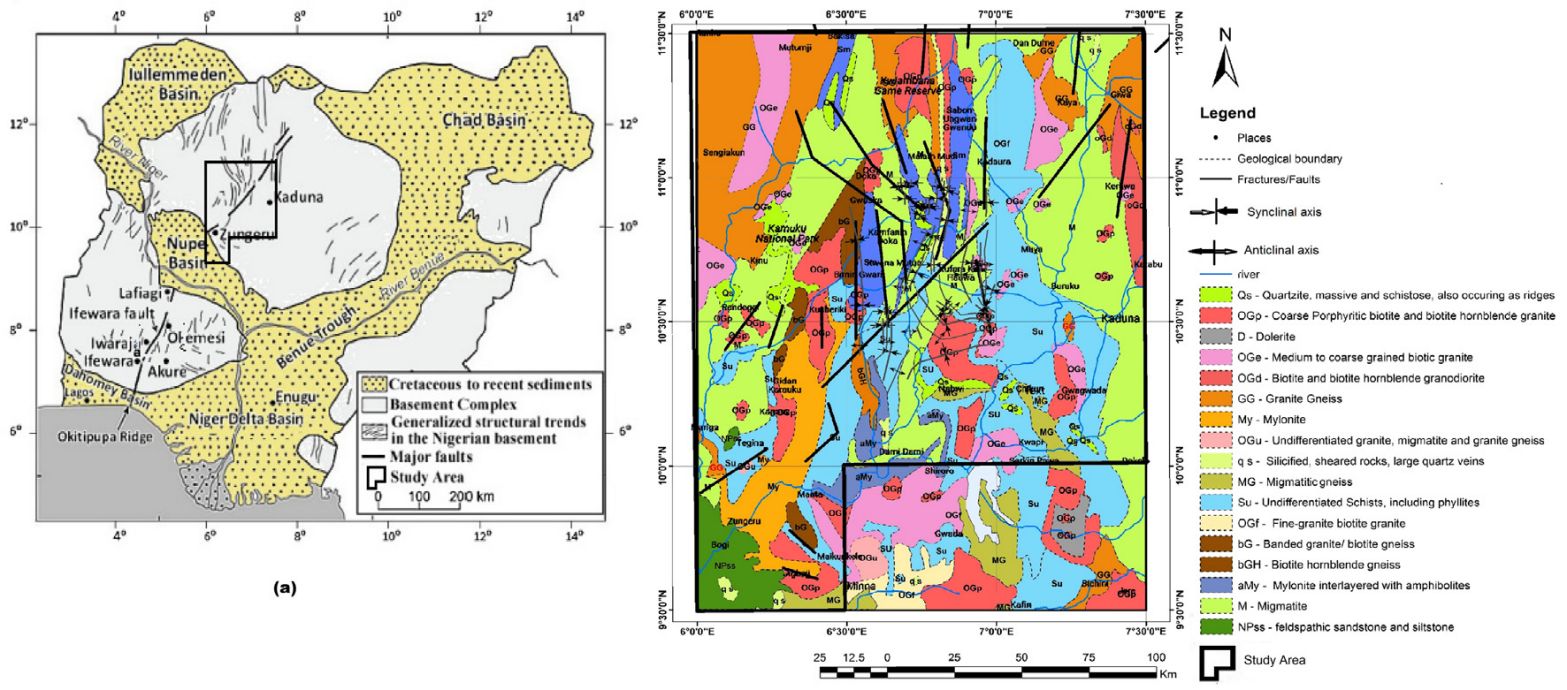
Geological map of (a) Nigeria showing the major faults (b) study area (modified from Truswell and Cope, 1963 in Garba, 2000; NGSA, 2004).

While the Zungeru mylonites are thought to have originated as a major thrust between basement (Kusheriki formation) and supracrustals (Birnin Gwari, Kushaka) (Wright et al., 1985), the Kushaka schist formation contrast with the Zungeru-Birnin Gwari belt in lithology, structure and igneous associations (Obaje, 2009). It is largely made up of fine-medium grained quartzo-feldspathic rocks interbedded with amphibolites and some quartzites (Dada, 2008). The Kushaka schist belt, which is known to host gold mineralisation, forms several curving schist belts, disjointed by the anticlines of gneiss. The study area's geological map is shown in Figure 1.

## 3 Methodology and data processing

### 3.1 Landsat OLI and SRTM data acquisition and processing

**Figure 2:**
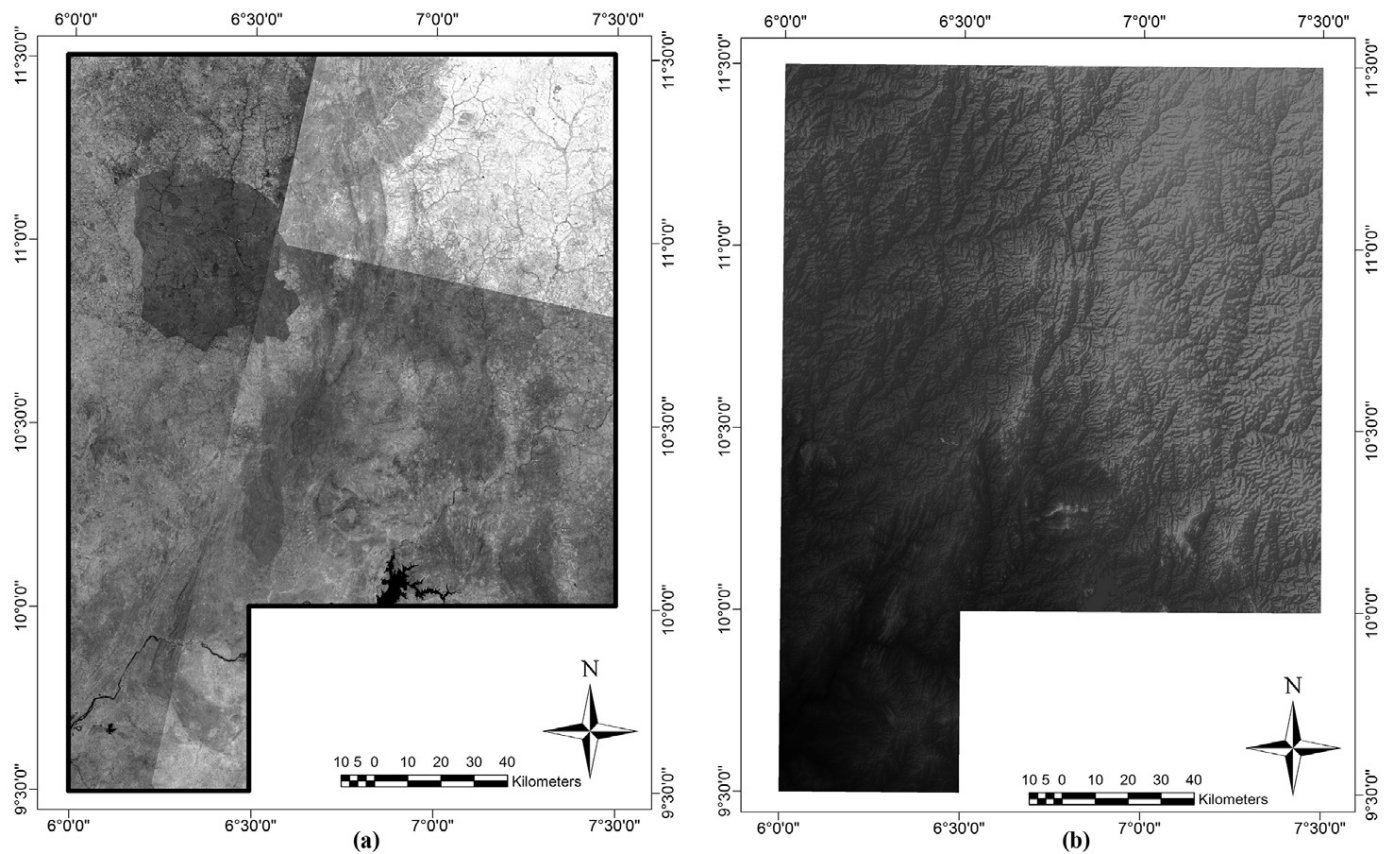
(a) Landsat-8 OLI and (b) SRTM-DEM imageries of the study area.

Landsat-8 OLI multispectral and Shuttle Radar Topography and Mapping mission (SRTM) imageries (Figs. 2a-b) were obtained from the USGS satellite database website. Landsat-8 OLI scenes 189–53/52 was acquired on 20 February 2020 while scenes 190–52/53 was acquired on 26^th^, January 2019. The Landsat-8 OLI imagery used was obtained during the dry season, when there was little or no atmospheric disturbance, which enhanced features with subtle physiographic expression and made the imagery more suitable for studying maximum surface anomalies. The raw Landsat-8 dataset was pre-processed with radiometric calibration, Fast Line-of-Sight Atmospheric Analysis of Spectral Hypercubes (FLAASH) atmospheric correction and image normalization. After pre-processing, the Landsat-8 OLI scenes were mosaicked to produce a single seamless image from which a subset covering the area of study bounded by Latitudes 9^o^ 30′ N - 11^o^ 30′ N and Longitudes 6^o^ 00′ E - 7^o^ 30′ E was extracted. To enhance the spatial characteristics, the Landsat-8 VNIR and SWIR bands (Ranganai and Ebinger, 2008; Fossi et al., 2021) with a 30 m pixel size were fused with a 15 m pixel size panchromatic band 8. In addition, the SRTM digital elevation data of the study area was hill-shaded using 30° light angle at azimuths of 0°, 45°, 90° and 135° to highlight E-W, NE-SW, N–S and NW-SE structural trends, respectively. The produced shaded-relief maps of the studied area will aid in revealing the influence of the subsurface structural features on the topography.

Automatic lineament's extraction was carried out using various value combinations for each LINE module's parameter of the PCI Geomatica software (Adiri et al., 2017; Shebl and Csámer, 2021). These parameters are RADI (filter radius), GTHR (Edge Gradient Threshold), LTHR (Curve Length Threshold), FTHR (Line Fitting Threshold), ATHR (Angular Difference Threshold), and DTHR (Linking Distance Threshold). The procedure was carried on the entire datasets to select the optimal parameter settings that resulted in appropriate output of the lineaments extracted. The extracted lineaments were validated by removing lineament errors such as those that align with the boundary of the subset satellite imageries and overlaying the lineaments on topographical maps and Google Earth image to remove false lineaments/curvilinear resulting from anthropogenic features (e.g. roads) of non-geological origin (Hung et al., 2005; Mallast et al., 2011; Akinluyi et al., 2018).

### 3.2 Aeromagnetic data acquisition and processing

The High-Resolution Aeromagnetic Data (HRAD) which covered parts of Niger, Kaduna, Katsina and Zamfara States were acquired from the Nigerian Geological Survey Agency (NGSA). The data were acquired between 2004 and 2009 by Fugro Airborne Survey Limited. The flight parameters of the HRAD are as follows: Flight line spacing – 0.5 km; Terrain clearance – 80 m; Flight lines – NW - SE; Tie line spacing – 2 km; Tie lines direction – NE - SW. The removal of IGRF was based on the epoch date of 1st of January 2005.

The HRAD were gridded at 100 m intervals using the minimum curvature gridding technique to produce a Total Magnetic Intensity (TMI) map of the study area. The study area lies within the magnetic equatorial regions of low inclination (I = 2.28^o^) where reduction to pole technique is not valid; since N–S bodies have non-detectable induced magnetic anomaly at zero geomagnetic inclination (Blakely, 1996; Grauch et al., 2004; Nabighian et al., 2005; Awoyemi et al., 2017b). The reduced to equator (RTE) filter was applied to the TMI grid to generate the RTE anomaly map which corrected the asymmetry associated with low-latitude anomalies (Blakely, 1996; Geosoft, 2015; Awoyemi et al., 2017a, 2017b; Arogundade et al., 2020). The RTE grid was subsequently upward continued to a height of 100 m to improve the signal-to-noise ratio and attenuate the noise/cultural features in the data.

Total Horizontal Derivative (THD) and Analytical Signal Amplitude (ASA) was applied to the filtered (upward continued) grid to enhance and delineate the signature of the subsurface structures and lithologic boundaries ((Nabighian, 1972; Blakely and Simpson, 1986; Roest et al., 1992; Thurston and Brown, 1994; Awoyemi et al., 2017b). The application of 3D Euler Deconvolution (ED) on the RTE grid using a structural index of one (1) was used to delineate the geometrical nature and depths of occurrence of the source edges (Reid et al., 1990). The resulting maps of THD, ASA and ED were integrated to produce a magnetic lineament map. A 2D forward model performed on the RTE grid involve the comparison of the calculated and observed magnetic data. The model was used to have a clear view of the subsurface structures and determine the magnetisation distribution within the source. The GM-SYS extension of the Oasis Montaj software was used to model the anomalies effect of the subsurface structures (e.g. Arogundade et al., 2020). In interpreting the observed data, eight profiles were taken along different parts of the RTE grid.

## 4 Results

### 4.1 Landsat-8 OLI and SRTM-DEM

**Figure 3:**
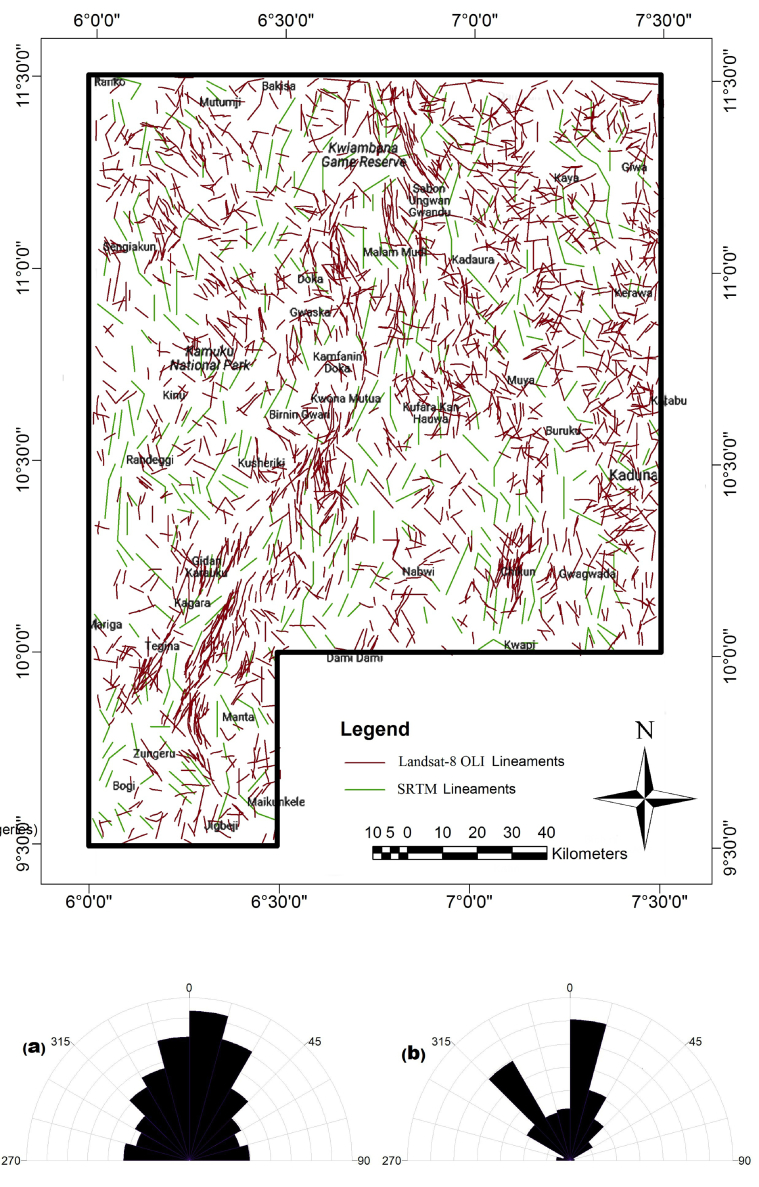
Lineaments derived from the satellite imageries (Landsat OLI and SRTM). Azimuth-frequency diagram for (a) Landsat-8 OLI (b) SRTM-DEM.

The extracted lineaments in Figure 3 show the cumulative outcome of the processed Landsat-8 OLI and SRTM imageries. The lineaments have been processed to show lineaments with the geologic origin, particularly recognising that not all the lineaments from satellite imageries have a magnetic signature. The Landsat-8 OLI lineament map clearly shows that the NE-SW trending lineaments from Zungeru to Birnin Gwari areas enclosed with Zungeru mylonites are dense (Kolawole and Anifowose, 2011). Also apparent is the concentration of lineaments evident in the southeastern parts, particularly around Chikun and the northern part around Kwaimbana and Doka, trending mostly in the NW-SE directions. The azimuth-frequency diagram derived shows that various lineament trends traverse the study area. The extracted lineaments from Landsat-8 show N–S, NNE-SSW, NW-SE, NNW-SSE, ENE-WSW and NE-SW trending lineaments while the lineaments extracted from the SRTM-DEM's shaded-relief maps are prominent in the N–S, NNE–SSW and NW-SE directions. The ample extent of the lineaments azimuths is due to the polycyclic migmatite, gneiss and granite terrain (Turner, 1983), which constitute the major lithology of the schist belt in the study area.

### 4.2 The Total Magnetic Intensity, RTE and filtered anomaly

**Figure 4:**
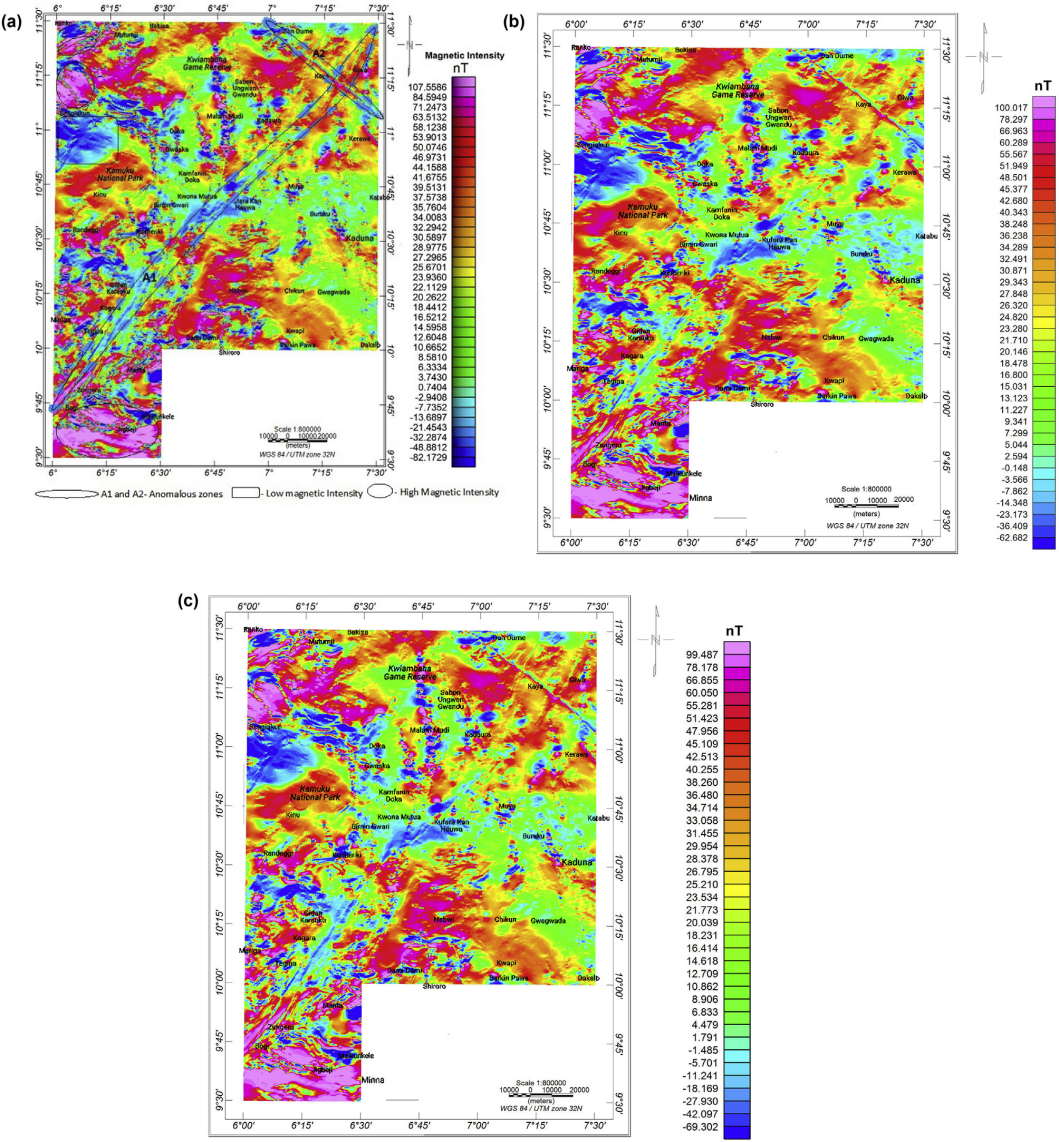
(a) Total magnetic intensity (TMI) map of the study area. (b) Reduced to equator (RTE) map of the study area. (c) Upward continuation map of the TMI to a height of 100 m.

The variations in the magnetic field intensity across the TMI, RTE, and filtered maps (Figs. 4a-c) are ascribed to the differences in magnetic mineral content between the different rock units and the varying depths of the underlying rocks (Figure 4a) (Oladele et al., 2016). Some of the areas observed with high magnetic intensity values are Jigbeji (lower part of the study area) with anomalies in the E-W direction and parts of Sengiakun and Mutumji (Northwest). More so, the E-W direction of the magnetic signatures is due to the location of the magnetic sources in low latitude (Reeves, 2005). Areas with a minimum magnetic intensity value of -7.73 to -82.2 nT are conspicuous around Sengiakun. Rocks characterised by the low intensity in low latitude areas (such as the study area) usually have high susceptibilities.

In contrast, rocks with a high magnetic intensity usually have low magnetic susceptibilities. The NE-SW trending linear anomalous zone extends from the Bogi-Zungeru (Southwest) to the Giwa area (the northeastern portion of the study area), while an elongated NW-SE trending anomaly was observed in the northeastern part of the study area. A qualitative examination of these conspicuous magnetic patterns revealed that the linear anomalous zones are reflected by magnetic lows bounded by magnetic highs and can be interpreted as reflecting an extensive structure in the Precambrian Basement. As discussed by Stoneley (1966), the NE-SW and NW-SE magnetic signatures are generally consistent with the tectonic trends of the West African Precambrian rocks. Also, an anomaly pattern cut through Malam Mudi and Kwaimbana (north central) with detectable positive-negative anomaly pairs representing folded north-south trending structures (Beard, 2000). This N–S structural imprint might have been initiated during the Pan-African orogeny (Olasehinde et al., 1990; Salawu et al., 2021).

### 4.3 Edge detection and structural mapping

**Figure 5:**
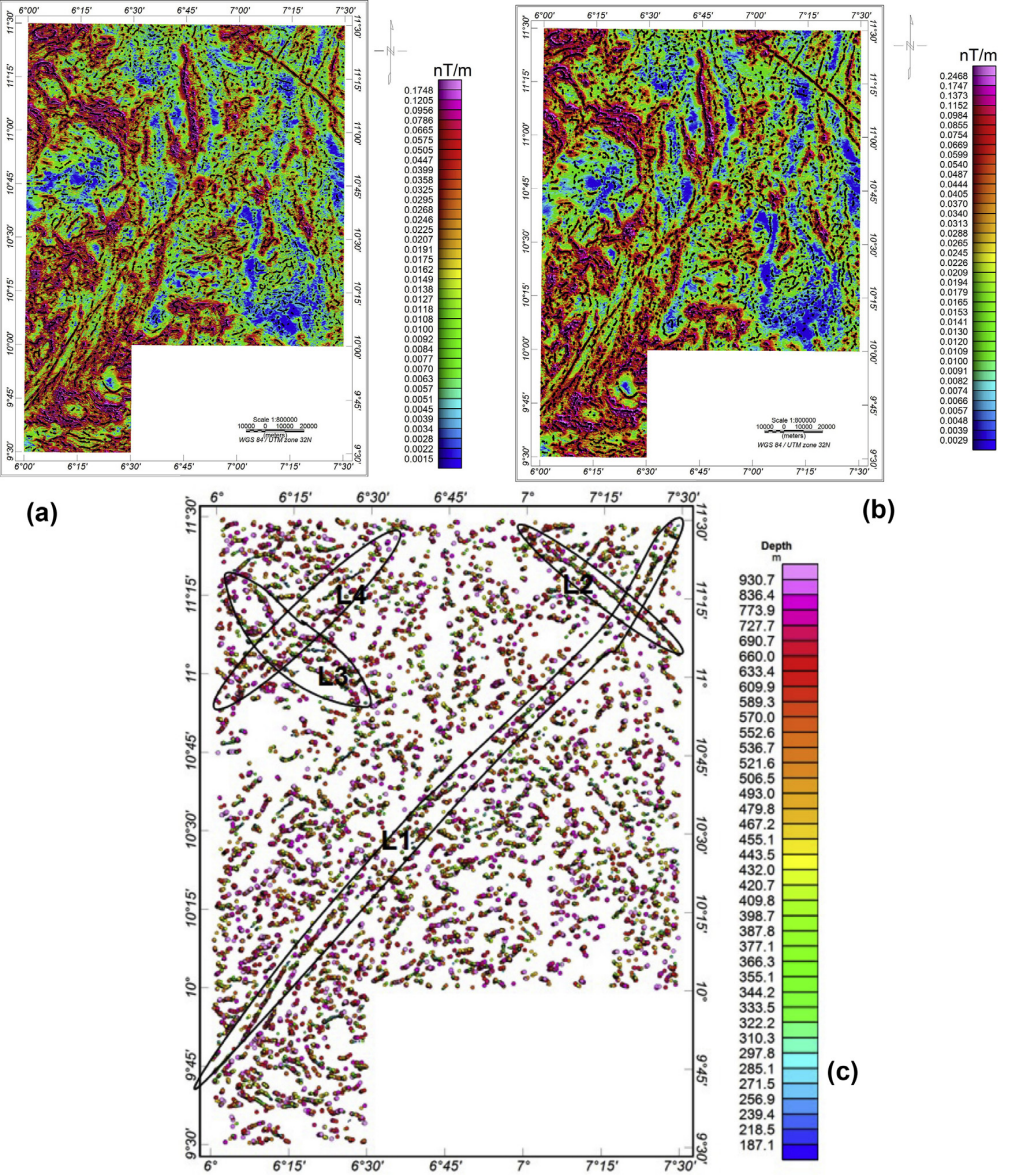
(a) Total horizontal derivative map with maximum amplitude points overlain on the map (b) Analytic signal amplitude map with maximum amplitude points overlain on the map (c) Euler solutions plot using structural index of 1 (depth varies with colour).

The peaks (i.e., the source edge locations) derived from THD and ASA was overlaid on the THD and ASA maps, respectively (Figures 5a and 5b) to delineate the contact locations and directions. As THD peaks over the source edges, so does ASA (Hosseini et al., 2013). The pattern of the linear anomalous zones on the THD and ASA maps are similar. It corresponds with the notable magnetic anomalies and shows prominent trends in the NE-SW and NW-SE directions, which is attributable to Pan African orogeny (600 Ma) and Kibaran Orogenic (1100 Ma) cycle of deformation (Burke and Dewey, 1972; Fitches et al., 1985; Dada, 2006).

Figure 5c shows the result of 3D Euler deconvolution (ED) for the study area. Reid et al. (1990) indicated that a suitable structural index (SI) is reflected with clustered solutions and notable anomalies. Reid et al. (1990) observed in synthetic models that a fault, dyke, or sill edge with a limited throw is best displayed with a SI of 1.0, while a SI of 0 best represented a large throw fault. However, he concluded that complex structures such as faults require slightly higher indices other than a SI of 0 which is used for contact. Therefore, to infer faults, their geometry and depths of occurrence within this study area, a SI of one (1) was selected for this study and gave well-clustered solutions. The Euler solutions show the spatial locations and non-uniform depths of the interpreted structures (Figure 5c). Depths ranging from 180 to over 900 m were distributed across the study area. Some important zones on the Euler plot are made noticeable by oval shapes (L1 to L4). L1 zone shows contiguous solutions with uneven depths approximately in NE-SW direction outstretch to the northeastern part from the southwestern part (Bogi-Zungeru) of the study area. It also served as conjugate pair to L2 solutions. L3 and L4 zones observed in the northwestern also form a conjugate pair. These solutions matched with the source edges that were also delineated with the ASA and THD methods.

**Figure 6:**
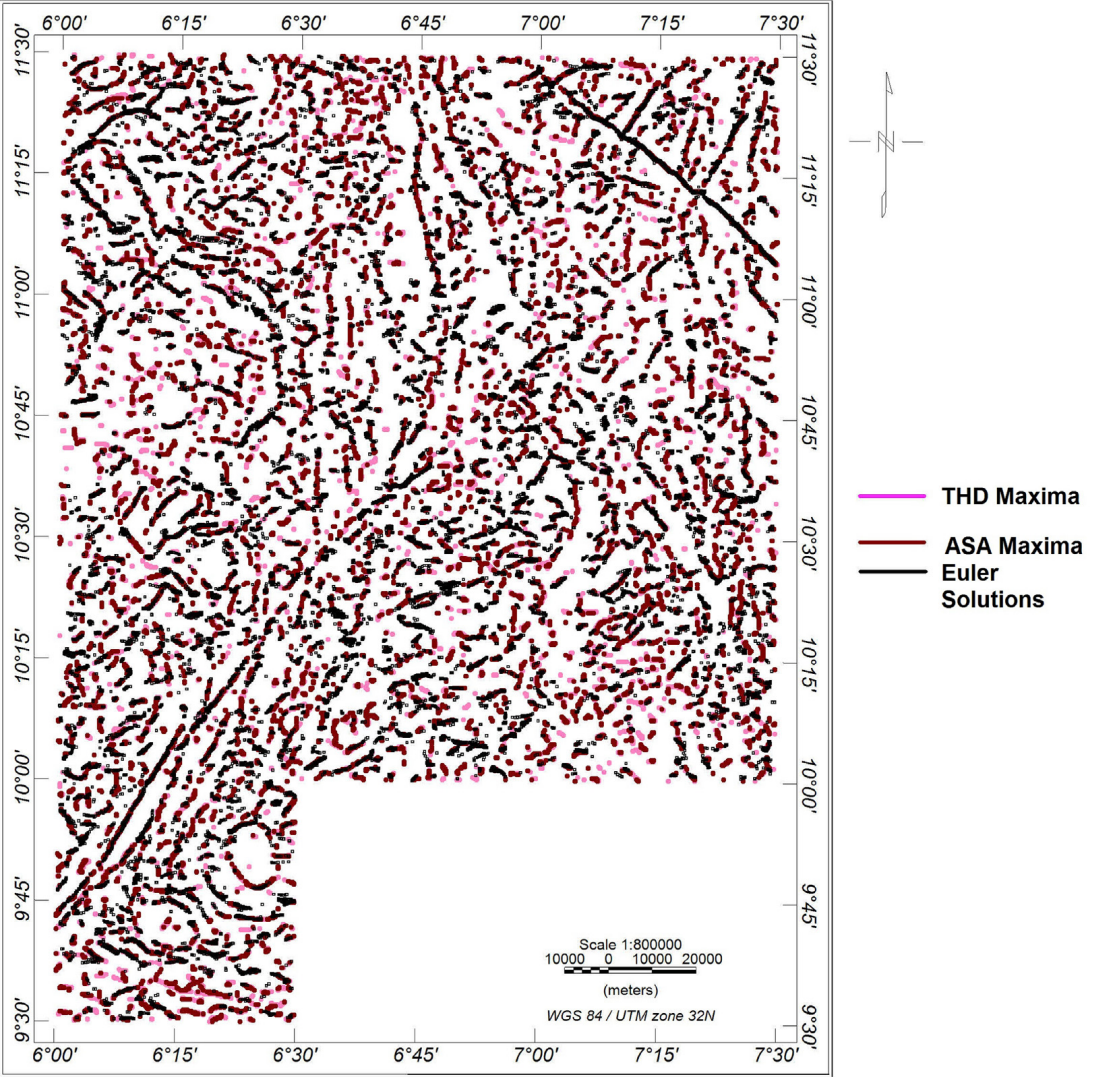
Composite map of the estimated locations from THD (pink), ASA (brown) and Euler solutions (black).

**Figure 7:**
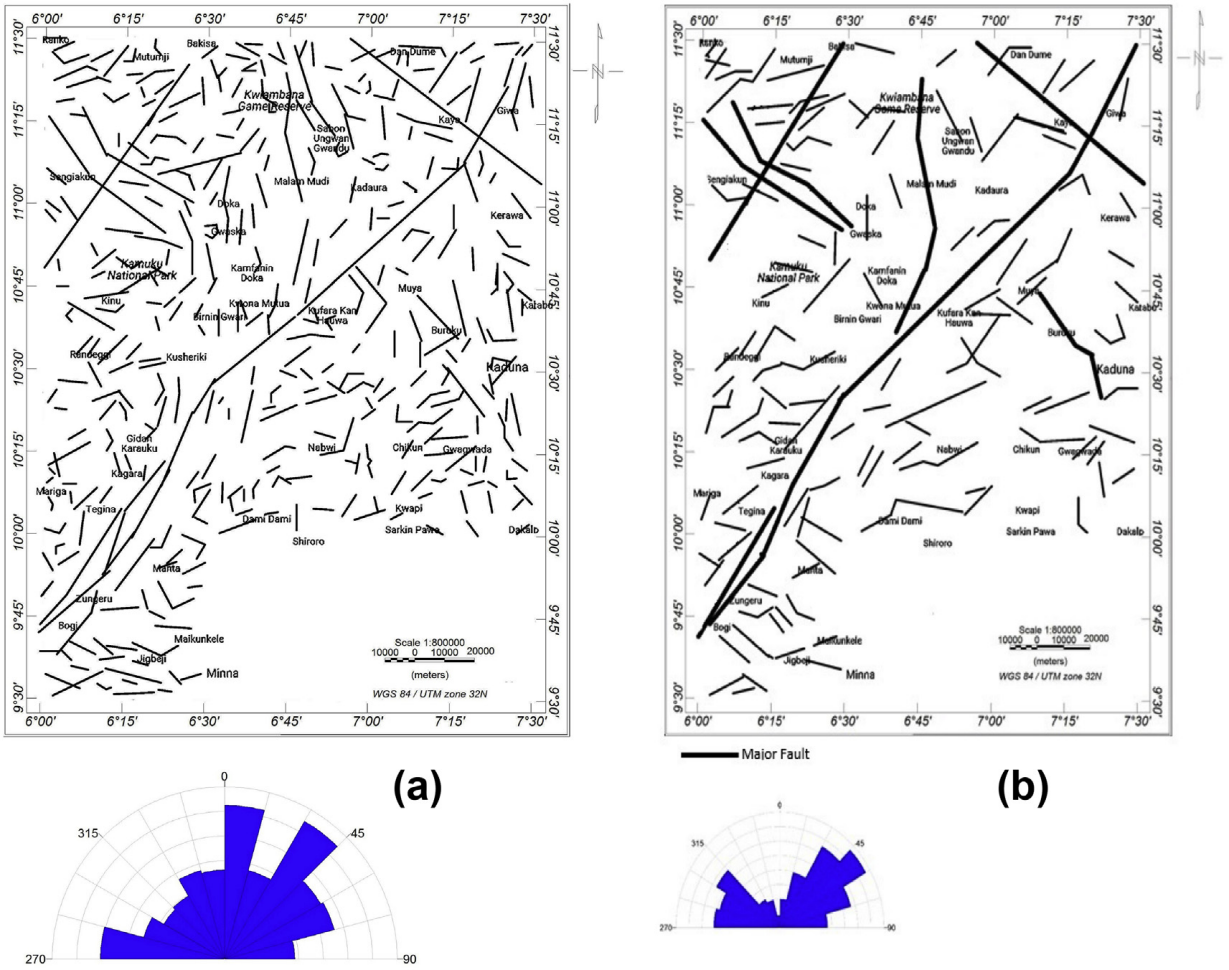
(a) Inferred contact locations map from THD and ASA (b) Lineament map of the study area showing the inferred faults from the composite map of the THD, ASA and Euler solutions.

The composite map of all the source edge detection techniques was produced by superimposing the maxima of ASA (brown), THD (pink) and Euler solutions (black) (Figure 6). The matching maxima/solutions of THD, ASA, and ED indicate subsurface rock boundaries which could be lithological contacts, faults or fractures (Figure 7). Apart from the reliability deduced from the use of more than one technique for locating the magnetic source edges, the conflation of these techniques has diverse significance on the trend, length, dip, depth of the subsurface structures, and subtle magnetic rocks that would have otherwise been hidden; thus, giving a high-resolution view of how the study area is structurally fragmented.

### 4.4 Magnetic Modelling

**Figure 8:**
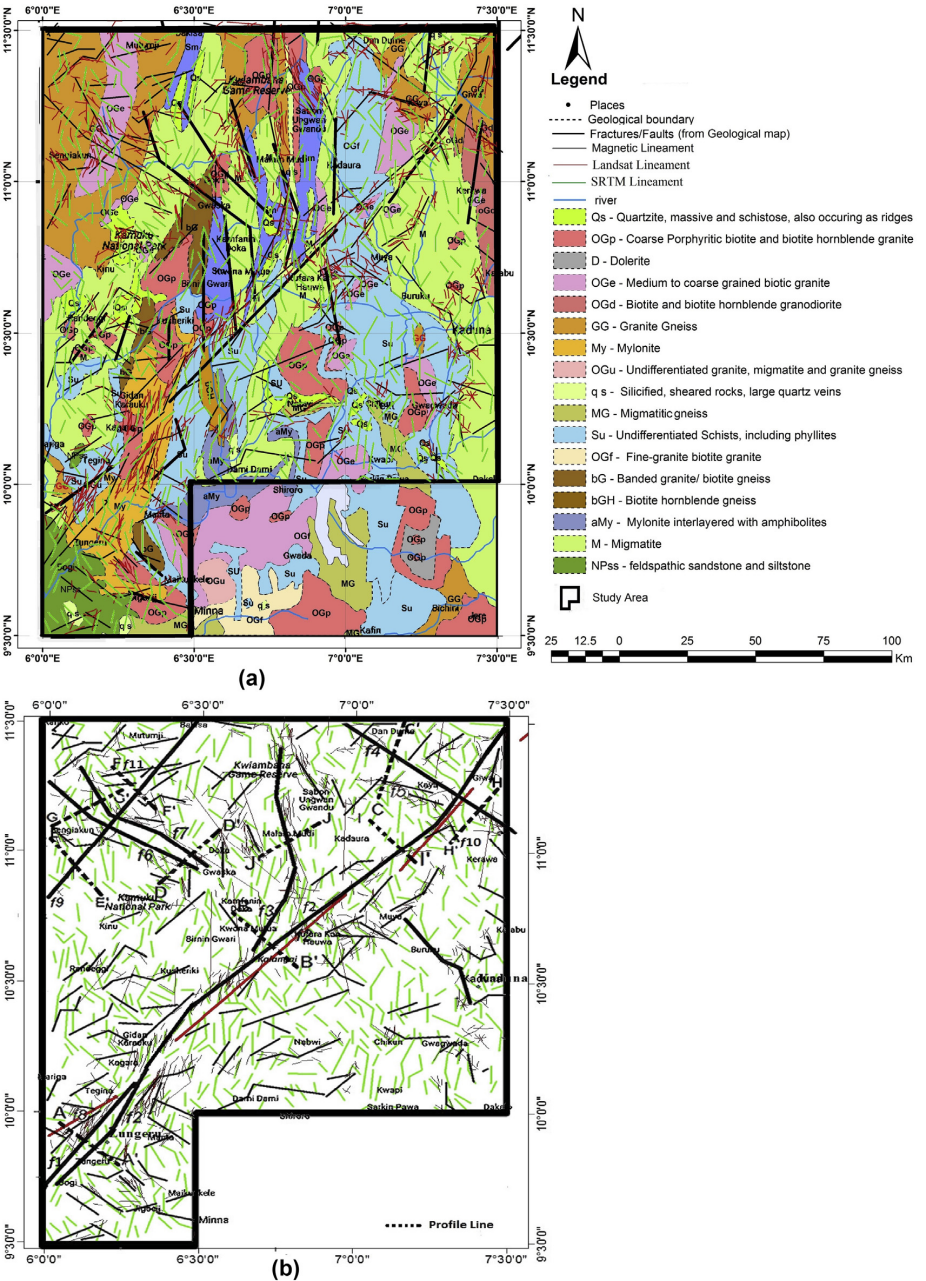
(a) Inferred structural map of the study area. (b) Magnetic profiles drawn across major faults.

2D magnetic modelling was performed to confirm the location of the faults, their dips and depth of occurrences in the area (e.g Arsè ne et al., 2019). The 2D forward modelling generates magnetic models that are similar to the observed field by modelling the basement topography with magnetic characteristics (Awoyemi et al., 2017b; Arogundade et al., 2020). Eight profiles were taken in directions orthogonal to the prominent inferred faults (Figure 8b). The Euler depth solutions were obtained across each profile, and depths from source parameter imaging were considered in creating the model. The susceptibility of the overburden within the study area is assumed zero. In contrast, the susceptibility values for the different basement blocks were varied to obtain a good fit in this modelling exercise. Aeromagnetic profiles across individual linear anomalies typically exhibit symmetric curves of the type expected over offset layers, as well as curves with two asymmetric peaks and two or more inflection points (Grauch et al., 2000). The structural or lithologic contacts could be represented by the contacts between the blocks at inflection points of the observed magnetic field on the profile (Hinze et al., 2013).

## 5 Discussion

The contact locations (Figure 7a) inferred from THD and ASA trend in various directions, dominantly in N–S, W-E and NE-SW. Other trends observed on the azimuth-frequency diagram are NNE-SSW, WNW-ESE, NW-SE, NNW-SSE and ENE-WSW directions. Across the study area, some contact lineaments extend several kilometres. These contacts may represent elongated boundaries or major faults between different rock units that might have been affected by the same tectonic history. The locations where the THD contacts offset or are slightly parallel from ASA contacts implies the down-dip direction with the true contact location(s) shown by ASA contact location(s). However, the contact location(s) is said to be vertical when both are overlapping each other (Philips, 2000; Awoyemi et al., 2017a; 2017b).

The structural map is composed of major and minor faults (Figure 7b). The faults inferred are derived from the combined structural analyses of the THD, ASA, and ED methods (Figure 6). The azimuth-frequency diagram shows the trending pattern in the NE-SW, ENE-WSW, E-W and NW-SE directions, among which the NE-SW trend dominates (Figure 7b). Other trends identified on the azimuth-frequency diagram are N–S, NNE-SSW and WNW-ESE directions. The tectonic process could have influenced similar patterns of the structures in the studied area (Awoyemi et al., 2017a).

Wright (1976) opined that the Chain and Romanche fracture zones could be linked to the prominent NNE-SSW and NE-SW shear zones of the southwestern Nigerian Basement. The shear zones correspond to the major faulting associated with the Northern Nigeria's Schist belt. These fracture systems' pattern was most likely established during the Pan-African Orogeny (McCurry, 1971). The NE-SW, ENE-WSW, N–S and NW-SE structural trends identified from satellite imageries and aeromagnetic maps can be taken as significant features of the tectonic framework in the Nigeria Basement Complex (Oluyide, 1988; Olasehinde et al., 1990). Moreover, Wright et al. (1985), Ananaba and Ajakaiye (1987), Caxito et al. (2020) revealed that the structural grain of the Precambrian rocks in most of West Africa generally lies between the N–S, NW-SE and NE-SW. The E-W structures appear to have been produced in earlier Orogeny events (pre-Pan-African Orogeny) (Salawu et al., 2021), consequent to the dominant Pan-African Orogeny.

The lineaments delineated from the HRAD and satellite imageries were superimposed on the existing faults shown on the published geological map of Nigeria for possible correlation (Figure 8). With the result presented from the composite map, several undetected lineaments traversing the study area were revealed. Some of the interpreted deeper structures (Figure 5c) correspond to multiple lineaments at the surface. However, a number of the structure have no surficial expression, which suggests they are buried faults. The lineaments are denser on the satellite imageries than on the HRAD because the datasets respond to different physical properties of the geological units and features. The difference in the physical properties of the different techniques is because the aeromagnetic dataset responds to susceptibility contrasts of rocks, while the satellite imageries rely on surface radiation reflectance.

From Figure 8, the location and orientation of the major fault labelled *f*2, which extend about 245 km, shows a close spatial correlation with the existing Zungeru/Kalangai transform fault zone on the published geological map of Nigeria. The Zungeru/Kalangai transform fault zone, as depicted on the structural map, runs from Nigeria's southern region to the study area (Awoyemi et al., 2017a; 2017b) and cuts through Bogi, Zungeru passing through Tegina, Kafura Kan Hauwa, Kadaura to Giwa and show continuation beyond the study area. The fracturing could have occurred as a response to the regional deformation from pre-existing zones of weakness, thus, propagating in an approximately NE-SW direction to the study area. According to Onyedim and Ocan (2001), the Ifewara-Zungeru/Kalangai shear zone most likey existed with the mylonites' emplacement at Ifewara and Zungeru areas, implying that the mylonites might have been produced as a result of Pan-African shearing along the Zungeru/Kalangai fault. McCurry (1971), Francheteau and Le Pichon (1972), Oluyide (1988) opined that the Zungeru transform fracture systems are continental projections of oceanic transform (transcurrent) faults, and the pattern of these fracture systems was probably established during the Pan-African orogeny. Studies have also shown that some tremors experienced in Nigeria might be associated with the dynamics of the oceanic fracture zones (e.g. Akpan and Yakubu, 2010; Afegbua et al., 2011; Shiwua, 2013; Tsalha et al., 2015; Thomas et al., 2020; Olugboji et al., 2021). These studies show that the epicentre of a reported occurrence of an earth tremor in Lupma, Niger State lies along with the northward extension of the Zungeru-Kalangai fault zone. However, these studies have revealed that there is no confirmation that the Ifewara-Zungeru-Kalangai transform fault is active at the moment.

Many other NE-SW trending faults in the study area could be associated with the deformation history of the Zungeru/Kalangai fracture system and are likely to have the same tectonic origin. Also observed on the structural map is the NW-SE trending fault (labelled *f*4) around Kaya, intersecting the Zungeru-Kalangai fault (*f*2) and creating a conjugate pair in the northeastern part. A NE-SW trending fault *f*9 around Sengiakun is bisected by faults *f*6 and *f*7 in the northwestern part of the study area. The NE-SW trending fault *f*9 could have resulted from the deformation history of the Zungeru-Kalangai fault zone, which is most likely to have the same tectonic origin.

**Figure 9:**
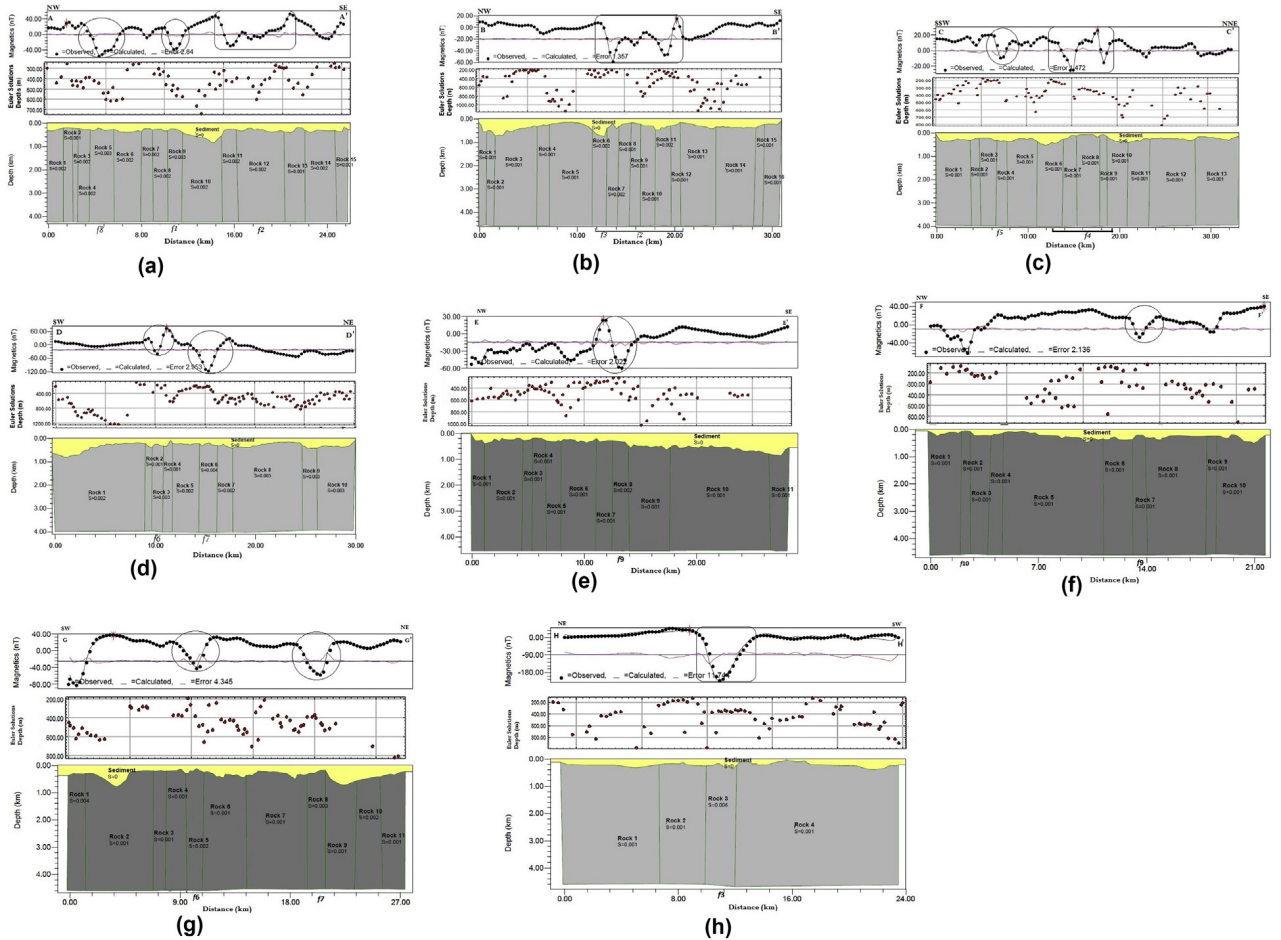
Geological model along (a) Profile A-A' (b) Profile B–B' (c) Profile C–C' (d) Profile D-D' (e) Profile E-E' (f) Profile F–F' (g) Profile G-G' (h) Profile H–H'.

The model from profile A-A′ was taken around Zungeru, southwestern part of the study area in the NW-SE direction and extended 25.7 km (Figure 8b). The observed anomalies along this profile ranged in magnetic intensity from -55.38 to 53.41 nT, while the Euler solutions along the profile have a depth range of 245.7–743.6 m. The anomaly in Figure 9a showed a magnetic response characterised by a low negative anomaly that occurs between high positive anomalies. For example, the shape of the anomaly shown with oval-shaped (blocks 5 and 9) on Figure 9a is a typical representation of a thin dyke which shows a relatively high susceptibility (0.003 cgs) than the surrounding blocks (0.002 cgs). In contrast, the anomaly marked by a rectangular-shaped (bounding blocks 11, 12 and 13) represents a thick dyke. Euler depth solutions complemented the model. The structures *f*1, *f*2 and *f*8 correspond to the block boundaries labelled in profile A-A'. The Zungeru-Kalangai fault zone *f*2, dipping SE, occurs at a depth range of 266–602 m. Also, smaller structures *f*1and *f*8 along the profile occur at a depth range of 373–619 m. According to Gettings (2005), Isaksson et al. (2007), the magnetic susceptibility of rocks reduces due to the alteration of rock from fractures. Thus, the low susceptibility observed within the area might be a result of fractured/faulted rock.

Profile B–B′ was taken in the NW-SE direction along Kwona Mutua and Kufura Kan Hauwa located at the central part of the study area. It extends a distance of 30.9 km, and the observed magnetic intensity along the profile ranged from -59.22 to 15.03 nT. This profile (Figure 9b) was modelled as sixteen (16) basement blocks whose boundaries are associated with structural features (e.g. contacts and faults). These contacts boundaries may be responsible for the uplifted blocks (horsts) and down-faulted blocks (graben). Blocks 6 to 12 are displaced by the effect from structures *f*2 and *f*3. The magnetic response in carved rectangular-shape between blocks 6 to 12 is characterised by low negative anomalies bounded by positive anomalies, representing a thick dyke. The Zungeru-Kalangai transcurrent fault zone *f*2 is dipping SE, as also confirmed by profile A-A'. The depth of the fault (*f*2) at this point ranged from 198 to 809 m. Profile C–C′ was taken along SSW to NNE direction around Dan Dume and Kaya in the northeastern part of the study area. This profile covers a distance of 32.9 km, as shown in Figure 9c. The presence of V-shaped anomalies and the clustered Euler depth solutions above blocks 6 to 10 and block 4 shows it is most likely to be a dyke. Blocks 6 to 10 are displaced by structure *f*4 and has a typical magnetic response of a thick dyke. The source location of *f*4 is complemented by Euler depth solutions which range between 218 and 447 m. A structure *f*5 along with the profile with a depth range between 195 and 467 m displaced at block 4 with a vertical dip.

Profile D-D′ covered a distance of 29.9 km and was taken along the SW-NE direction, passing through parts of Dan Dume and Giwa in the eastern part of the study area (Figure 9d). This area was modelled as ten (10) blocks with susceptibility values ranging from 0.001 to 0.004 cgs. Blocks 3 (0.003 cgs) and 7 (0.004 cgs) show a magnetic response of a thin dyke with a relatively high susceptibility than the surrounding blocks and are displaced by two major structures *f*6 and *f*7, which are both dipping SW. The source depth obtained for *f*6 ranged from 176 to 451 m while *f*7 ranged from 191 to 665 m.

Profile E-E′ was taken in the NW-SE direction around the northwestern part of the study area. This profile (Figure 9e) was modelled as eleven (11) basement blocks and extended a distance of 28.5 km. The profile was complemented by Euler depth solutions which ranged from 231.23 to 1021.9 m. Block 8, which fault *f*9 displaces, shows a relatively high susceptibility of 0.002 cgs than the surrounding blocks (0.001). Fault *f*9 have a vertical dip. The source location of *f*9 ranged from 231 to 603 m.

The magnetic profile F–F′ was taken in the NW-SE direction in the northwestern part of the study area and covered a distance of 21.6 km. This profile (Figure 9f) was modelled as ten (10) basement blocks. Block 2 is displaced by a structural feature f10, while block seven is displaced by f9. The magnetic response from both structures is a typical representation of a dyke. The depth of f9 at this location occurs from 120 to 340 m.

Profile G-G′ was taken across faults *f*6 and *f*7 from SW to NE in the northwestern part of the study area. This profile covers a distance of 27.5 km, as shown in Figure 9g. Judging by the presence of the V-shaped anomalies above blocks 5 and 8 displaced by structural features f6 and f7, it is most likely a thin dyke. These conformed to the structural features earlier explained on profile D-D'. The susceptibilities of blocks 5 and 8 are 0.002 and 0.003 cgs, respectively which is relatively high than the surrounding blocks. The depth of f6 and *f*7 ranged from 191 to 655 m and 368–547 m, respectively.

Profile H–H′ was taken across the upper part of structure *f*3. This profile covers a distance of 23.5 km, as shown in Figure 9h. Profile H–H′ was modelled as four (4) blocks with susceptibility ranging from 0.001 to 0.006 cgs. Block 3 is displaced by a structure *f*3 whose susceptibility is higher (0.006 cgs) than the surrounding blocks (0.001 cgs). The shape of the anomaly above block three and its high susceptibility indicate it is a dyke as confirmed by profile B–B'.

The 2D models of the basement blocks along the selected profiles show the structures, their approximate source locations and dips (Figs. 9a-h). They agree with the application of THD, ASA, and ED methods to the HRAD. The results revealed the existed Zungeru-Kalangai fault zone and other several hitherto undetected structures such as *f*3 (northern part of the study area), *f*4 (located around Kaya in the northeastern part of the study area), *f*6 and *f*9 (located around the Sengiakun area in the northwestern part of the study area). The structural attributes of some of the inferred faults delineated are displayed in Table 1.

**Table 1:** Attributes of some of the Inferred Faults.

Inferred Fault Segment	Strike	Dip
*f*1	NE-SW	NW
*f*2	NE-SW	SE
*f*3	NW-SE	VERTICAL
*f*4	NW-SE	SW
*f*5	WNW-ESE	VERTICAL
*f*6	NW-SE	SW
*f*7	NW-SE	SW
*f*8	NE-SW	NW
*f*9	NE-SW	VERTICAL
*f*10	NW-SE	NW

## 6 Summary and conclusion

Integrated satellite imageries and High-Resolution Aeromagnetic Data (HRAD) have been employed to reveal undetected structures and buttress the extension of the Zungeru-Kalangai fault zone from the Bida Basin in Nigeria. To delineate the possible locations, depths, strikes, dip directions of the structural features within the study area, the geologic lineaments from the remotely sensed data and magnetic lineaments from the aeromagnetic data were analysed and interpreted. The lineaments trend on the satellite imageries showed a prominent lineament trend in N–S, NE-SW and NNE-SSW directions. The lineaments trending in the NE-SW direction are dense around the Zungeru, Tegina and Gidan Karauku area, enclosing the mylonites that might have been produced due to Pan-African shearing. The identified structural trends on the HRAD strike N–S, NNE-SSW, NW-SE, ENE-WSW, E-W, NE-SW, and WNW-ESE. The orientation (NE-SW) and location of the inferred fault emanating from the Bogi-Zungeru area (southwestern) to the Giwa area (northeastern) on the magnetic lineament map suggest a significant fault in the study area. This major fault was bisected by the NW-SE fault, thereby creating a conjugate pair on the fault at the Kaya area (northeastern part).

Comparing the extracted structures from the HRAD and satellite imageries with the existing faults digitised from the geological map of Nigeria showed a significant correlation between the location and orientation of the Zungeru-Kalangai transform fault zone. Many other NE-SW trending faults within the study area could be associated with the deformation history of the Zungeru/Kalangai fracture system. Close lineaments in the same orientation/direction with the Zungeru/Kalangai fault show they are likely to have the same tectonic origin. The fracturing might have resulted as a response to the regional deformation propagating through the study area in an approximately NE-SW direction. The surface lineaments are denser on the Landsat OLI and SRTM imageries than on the HRAD because the datasets respond to different physical properties (surface radiation reflectance and susceptibility contrast of rocks) of the geological units. Eight profiles were selected across the anomalies reflecting prominent structures and modelled to reveal the 2D image of the basement fault blocks and depths. The variations in magnetic susceptibility of the basement rocks were revealed, and low magnetic susceptibilities of rocks observed on the models were due to the fractured bedrock within the study area.

The Zungeru-Kalangai transform fault zone dip SE and form a conjugate pair with a fault around the Kaya area. It was observed that the transform fault zone propagated into the study area through the Bida Basin Basement. The presence of several major and minor structural features with and without surface expression corresponding to some unknown and known faults was revealed with the combined interpretation of satellite imageries and HRAD. This has provided more details, lacking in previous geological maps.

## Declarations

### Author contribution statement

A.B. Arogundade: Conceived and designed the experiments; Performed the experiments; Analyzed and interpreted the data; Contributed materials, analysis tools or data; Wrote the paper.

M. O. Awoyemi: Conceived and designed the experiments.

O. S. Hammed, S. C. Falade & O. D. Ajama: Contributed materials, analysis tools or data.

### Funding statement

This research did not receive any specific grant from funding agencies in the public, commercial, or not-for-profit sectors.

### Data availability statement

Data will be made available on request.

### Declaration of interests statement

The authors declare no conflict of interest.

### Additional information

No additional information is available for this paper.
